# Prolonged Impella 5.0/5.5 support within different pathways of care for cardiogenic shock: the experience of a referral center

**DOI:** 10.3389/fcvm.2024.1379199

**Published:** 2024-07-02

**Authors:** Marina Pieri, Alessandro Ortalda, Savino Altizio, Luca Bertoglio, Pasquale Nardelli, Evgeny Fominskiy, Elisabetta Lapenna, Silvia Ajello, Anna Mara Scandroglio

**Affiliations:** ^1^ Department of Anesthesia and Intensive Care, IRCCS San Raffaele Scientific Institute, Milan, Italy; ^2^ School of Medicine, Vita-Salute San Raffaele University, Milan, Italy; ^3^ School of Medicine, Brescia University School of Medicine, Brescia, Italy; ^4^ Department of Cardiac Surgery, IRCCS San Raffaele Scientific Institute, Milan, Italy

**Keywords:** mechanical circulatory support, cardiogenic shock, Impella, myocardial recovery, LVAD

## Abstract

**Aims:**

Impella 5.0 and 5.5 are promising low-invasive left ventricle (LV) temporary mechanical circulatory supports (tMCS) for cardiogenic shock due to LV mechanical unloading and are paired with powerful hemodynamic support. This study aimed to analyze data and destinies of patients supported with Impella 5.0/5.5 at a national referral center for cardiogenic shock and to assess the parameters associated with myocardial recovery and successful weaning.

**Methods:**

A single-center observational study was conducted on all patients treated with Impella 5.0 or 5.5 from March 2018 to July 2023.

**Results:**

A total of 59 patients underwent Impella 5.0/5.5 implantation due to profound cardiogenic shock, with acute myocardial infarction being the most frequent cause of shock (42 patients, 71%). The median duration of Impella support was 13 days (maximum duration of 52 days). Axillary cannulation was feasible in almost all patients, and 36% were mobilized during support. A total of 44 patients (75%) survived to the next therapy/recovery: 21 patients experienced recovery and 15 and 8 were bridged to long-term LVAD and heart transplantation, respectively. The global survival rate was 66%. The predictors of native heart recovery at multivariate analysis were the number of days on tMCS before upgrade to Impella 5.0/5.5 [hazard ratio (HR) 0.68 (0.51–9) *p* = 0.0068] and improvement of LVEF within the first 7–10 days of support [HR 4.72 (1.34–16.7), *p* = 0.016].

**Conclusions:**

Transcatheter systems such as Impella 5.0/5.5 revolutionized the field of tMCS. Myocardial recovery is the primary clinical target. Its prognostication and promotion are key to ensure the most proficuous course for each patient from cardiogenic shock to long-term event-free survival.

## Introduction

Patients with profound cardiogenic shock still experience high in-hospital mortality (1): whatever the cause, the more severe the level of shock at the time of treatment, the higher the mortality risk (1, 2). Mechanical circulatory support (MCS) has gained wide application for the treatment of cardiogenic shock (3–6), since it allows immediate restoration of tissue perfusion (7) and received a Class IIA recommendation by the European Society of Cardiology guidelines on heart failure (8). In patients with cardiogenic shock [Class C, D, and E of Society for the Cardiovascular Angiography and Interventions (SCAI) classification], MCS is basically aimed at keeping the patient alive in the acute phase and supporting the heart toward the next steps of treatment of the primary disease (9). Transaortic temporary ventricular assist devices introduced an innovation in the armamentarium of short-term MCS and consequently in clinical practice, since they enable mechanical LV unloading, through a low-invasive approach (9). Impella 5.0 and 5.5 devices (Abiomed Inc., Danvers, MA, USA) are powerful pumps in terms of unloading efficacy and forward flow. The axillary access (5, 10) allows survival to the acute phase of shock and support through different possible pathways of care, eventually up to several weeks (11–18). Furthermore, they allow respiratory weaning, oral feeding, and mobilization while still on MCS (19). Although Impella 5.0/5.5 has opened new perspectives in the treatment of CS, in terms of survival and candidacy to definitive therapy, the different pathways of care in the context of different etiologies of shock deserve specific investigation. In particular, full hemodynamic support with Impella 5.0/5.5 is expected to promote myocardial recovery (20), but the factors to be evaluated for the process of myocardial recovery after MCS with Impella 5.0/5.5 have never been described. Myocardial recovery in terms of sustained absence of heart failure symptoms after MCS weaning and after hospital discharge is a primary target for acute heart failure treatments (21). Early stratification of patients with a high chance of myocardial recovery and successful MCS weaning might influence clinical practice and help optimize the steps of support and their timing.

This study aimed to analyze data, outcomes, and pathways of care of patients with prolonged Impella 5.0/5.5 support at a national referral center for cardiogenic shock and MCS and to assess the rate and potential predictors, if any, of myocardial recovery.

## Materials and methods

This observational single-center study included all consecutive patients who were treated with Impella 5.0/5.5 device due to acute severe cardiogenic shock in the Cardiac Intensive Care Unit of IRCCS San Raffaele Scientific Institute (Milan, Italy) from March 2018 (first device implanted) to July 2023.

The study is in compliance with current guidelines for human studies and was conducted ethically in accordance with the World Medical Association Declaration of Helsinki and ISHLT Ethics Statement. The study was conducted with the approval of the ethical committee (retrospective observational study TP INN approved by San Raffaele Hospital Ethical Committee and last amended in September 2023). In-hospital records and echocardiographic images were evaluated to retrieve baseline patient characteristics and hemodynamic data, Impella support characteristics, length and complications, and data outcomes. Coronary angiography records were also *de novo* examined by an in-house interventional cardiologist blinded to the study. Data from all patients were anonymized and stored in an electronic database.

**Figure 1:**
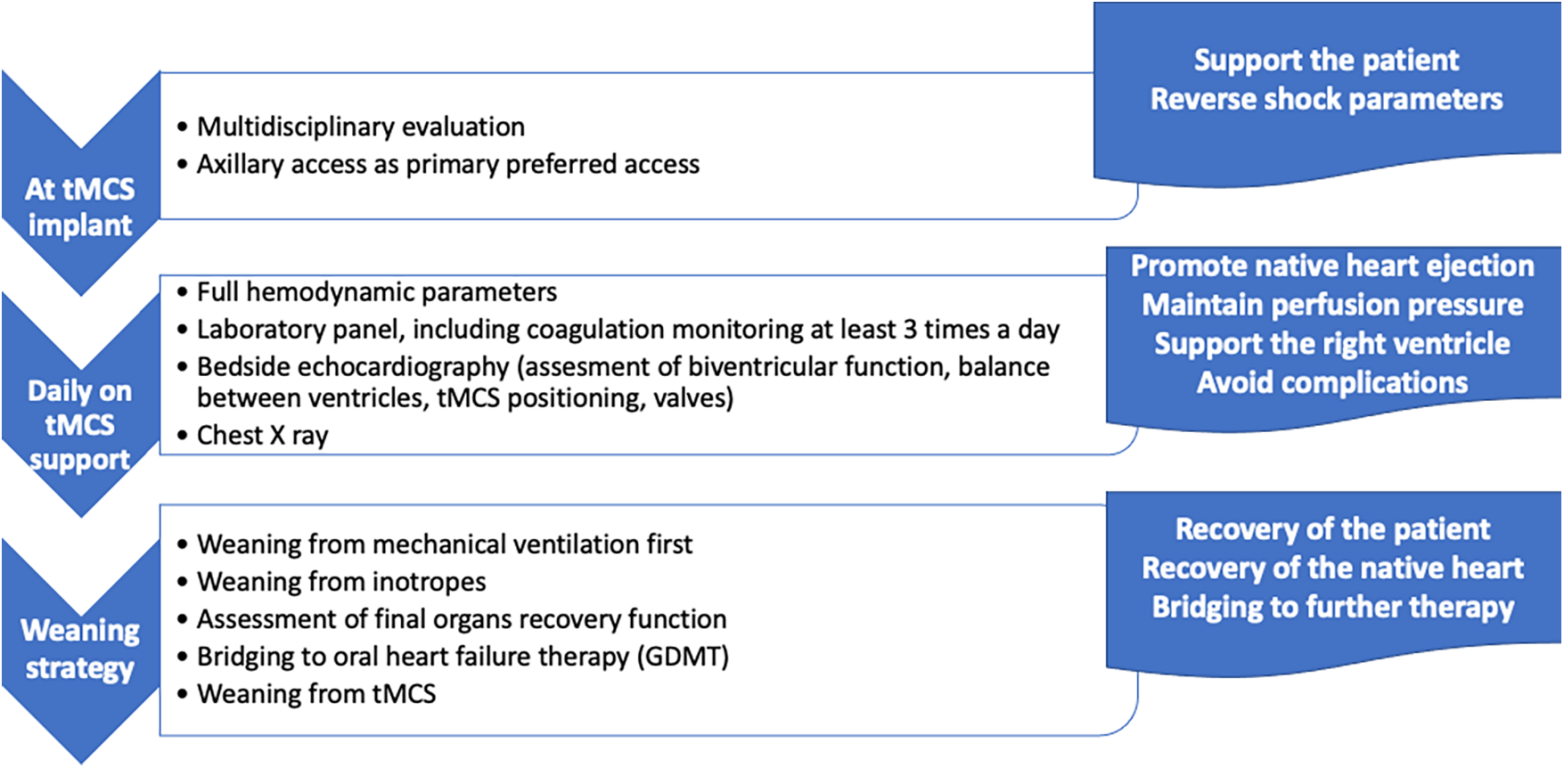
Flowchart of the management of patients on tMCS with Impella 5.0/5.5: steps and goals.

All patients were treated by the multidisciplinary heart team of our institution according to a standardized protocol (Figure 1). Impella 5.0/5.5 was implanted in the following clinical scenarios of cardiogenic shock, according to the clinical presentation of patients: (1) as first MCS device, in case of severe cardiogenic shock at presentation refractory to inotropes and medical therapy; (2) as “escalation therapy” in patients already supported with Impella 2.5/CP due to persistence of shock; and (3) as “de-escalation therapy” in patients on VA-ECMO to provide durable LV support and allow weaning from VA-ECMO. The patients resuscitated with ECLS after prolonged out-of-hospital cardiac arrest were considered for Impella 5.0/5.5 implantation only after neurological status assessment. The decision to implant the device and evaluation of MCS strategies were discussed within the multidisciplinary MCS team, staffed in the ICU, which included intensivists, cardiologists, and cardiac and vascular surgeons. Impella 5.0/5.5 was surgically implanted through axillary access as previously described (10). Daily monitoring encompassed full hemodynamic parameters and transthoracic or transesophageal echocardiography to optimize device performance and associated heart failure medical therapies to reach patient-tailored hemodynamic goals. All patients were administered systemic anticoagulation with bivalirudin with activated partial thromboplastin time of 55–60 s, according to our institutional protocol on anticoagulation during MCS (22, 23) plus antithrombotic agents if indicated. The patients received inotropic support prior to MCS implantation and during the initial phase of Impella support to promote residual native ejection, to maintain adequate mean arterial pressure (at least 70 mmHg), or to support the right ventricle. After Impella 5.0/5.5 implantation, the inotropic load was reduced and stopped as soon as possible and days before MCS weaning. Adverse events were recorded according to the most recent recommendations concerning short-term MCS (24). The patients with acute myocardial infarction (AMI)-related CS received primary percutaneous coronary intervention (PCI) at the in-house cath lab consistently with our institutional protocol. “Late presentation” refers to the presence of symptoms even on the days before the emergency department admission of the patient. Impella weaning was tested once the patients were hemodynamically stabilized, with normal extracardiac organ function and in the presence of pulse pressure with progressive reduction of *P* level and no inotropes. Mechanical ventilation and extubation were attempted before MCS weaning. Occasionally during MCS weaning (if tolerated), or after weaning, the patients were bridged to full heart failure medical therapies, including angiotensin receptor neprilysin inhibitor (ARNI), and were regularly followed up at the dedicated outpatient department after hospital discharge. In line with scientific literature (21), myocardial recovery was defined in terms of sustained resolution of the hemodynamic changes leading to shock sufficient to allow MCS explant (with the absence of heart failure symptoms, no inotropic support even after MCS weaning) and subsequently after hospital discharge on heart failure therapy. In the absence of hemodynamic recovery, patients were candidates for heart transplantation or LVAD implantation.

### Statistical analysis

Data were primarily stored in Excel (Microsoft, Redmond, WA, USA, Version 2103). The categorical variables are reported as numbers (percentage), and the continuous variables are reported as mean ± standard deviation (SD) or median (interquartile range) as appropriate, according to the Kolmogorov–Smirnov test. Group comparison was performed with a paired Student’s *t*-test or Mann–Whitney *U* test for continuous variables. Dichotomous data were tested with the *χ^2^* test or Fisher’s exact test, where appropriate. A *p-*value < 0.05 was considered a statistically significant difference between groups. A comparison between patients weaned from Impella due to native heart recovery vs. patients without myocardial recovery who could not be weaned from Impella was performed for all the variables under study. Odds ratios (OR) were estimated using logistic regression models in the overall sample. A multivariable logistic regression model was fitted to assess predictors for recovery, and covariates with univariable *p* < 0.10 were included. A backward variable selection algorithm with a stay criterion of 0.05 was applied, and for covariates in the multivariate model that violated the normality assumption, a logarithmic transformation was applied to normalize their distribution.

In the time-to-event analyses, a hazard ratio was calculated from the overall group populations using a univariate regression model by Fine and Gray. The main outcome “recovery” was analyzed by considering mortality, heart transplantation (HTx), and implementation of a left ventricular assist device (LVAD) as competing events. Covariates with univariable *p* < 0.10 were included in the multivariable Fine and Gray model.

Two-sided *p*-values <0.05 were considered significant. Statistical analyses were performed using SAS (Statistical Analyses System Inc., Cary, NC, USA, release 9.4) and the R (R Foundation for Statistical Computing, Vienna, Austria, release 4.2.3) software.

## Results

A total of 56 patients underwent MCS with Impella 5.0 and 3 patients with Impella 5.5 during the study period: 51 (87%) were male, with a median age of 60 ± 12 years (Table 1). As shown in Table 2, all patients were admitted with the most severe degrees of shock: 53 (90%) were INTERMACS Class 1 and 6 (10%) INTERMACS Class 2; 22 (37%) patients were in SCAI Class D and 37 (63%) in SCAI Class E; 16 (27%) had been resuscitated following cardiac arrest; almost all (57 patients, 97%) were on mechanical ventilation. The predominant cause of shock was acute coronary syndrome (42 patients, 71%), followed by acute severe decompensation of chronic heart failure (5 patients, 9%), chronic coronary disease (4, 7%), arrhythmic storm with ventricular tachycardia in dilatative cardiomyopathy (3 patients, 5%), postcardiotomy shock (3, 5%), and fulminant myocarditis (2 patient, 3%). Left ventricular ejection fraction was severely impaired [10 (0–20) %]. A total of 53 patients (90%) were already on temporary MCS at the time of the decision of Impella 5.0/5.5 implant: 14 patients had VA-ECMO + Impella 2.5 or CP device, 3 patients had VA-ECMO + IABP, 4 patients had VA-ECMO alone, 22 patients had Impella 2.5 or CP device alone, and 10 patients had IABP alone. A degree of end-organ dysfunction was already present at the time of Impella 5.0/5.5 implantation: creatinine was 2 (1.2–3.1) mg/dl and bilirubin 1.1 (0.9–1.6) mg/dl. Indeed 22 patients (36%) received renal replacement therapy during Impella support, and blood purification devices were adopted in 28 patients (50%). Revascularization procedure data are presented in Table 3. A total of 37 patients (88%) received coronary stenting, being the left main/proximal left anterior descendent as the culprit lesion in most cases (38 patients, 90%).

**Table 1:** Patients’ demographic characteristics.

	Total population (*n* = 59)	Recovery (*n* = 21)	No recovery (*n* = 38)	*p*-value
Males, *n* (%)	51 (87)	19 (90)	32 (84)	0.5
Age, years	60 ± 12	63 ± 10	59 ± 13	0.2
BMI	27 ± 4	28 ± 3	27 ± 4	0.5
Hypertension, *n* (%)	34 (58)	13 (62)	21 (55)	0.6
Diabetes, *n*	22 (37)	6 (30)	16 (42)	0.3
Chronic kidney failure, *n* (%)	7 (12)	3 (15)	4 (11)	0.7
Cerebral vascular disease, *n* (%)	1 (2)	1 (5)	0 (0)	0.4
Peripheral vascular disease, *n* (%)	7 (12)	3 (15)	4 (11)	0.7
Smoker, *n* (%)	19 (32)	7 (33)	12 (32)	0.9
COPD, *n* (%)	1 (2)	0 (0)	1 (3)	0.99
History of coronary artery disease, *n* (%)	26 (44)	10 (48)	16 (42)	0.7
Chronic heart failure, *n* (%)	20 (34)	4 (19)	16 (42)	0.09

Data are reported as median (interquartile range) or number (percentage), as appropriate.

BMI, body mass index; COPD, chronic obstructive pulmonary disease.

**Table 2:** Baseline data at Impella 5.0/5.5 implantation time.

	Total population (*n* = 59)	Recovery (*n* = 21)	No recovery (*n* = 38)	*p*-value
Cardiac arrest, *n* (%)	16 (27)	6 (29)	10 (26)	0.9
Acute coronary syndrome, *n* (%)	42 (71)	16 (76)	26 (68)	0.5
- STEMI, *n* (%)	39 (93)	16 (100)	23 (89)	0.2
- High risk NSTEMI, *n* (%)	3 (7)	0 (0)	3 (11)	0.5
Worst inotropic score	20 (11–30)	20 (13–30)	20 (13–30)	0.7
Mechanical ventilation, *n* (%)	57 (97)	20 (95)	37 (97)	0.7
Any MCS pre-Impella, *n* (%)	53 (90)	18 (86)	35 (92)	0.4
MCS pre-Impella, days	2 (1–5)	2 (1–2)	3 (1–7)	0.02
VA-ECMO, *n* (%)	19 (32)	7 (33)	12 (32)	0.9
VA-ECMO pre-Impella, days	2 (1–5)	2 (1–2)	5 (2–11)	0.02
Other Impella device, *n* (%)	37 (63)	11 (52)	26 (68)	0.2
Other Impella device, days	4 (2–7)	2 (2–2)	5 (2–8)	0.051
INTERMACS Class 1, *n*(%)	53 (90)	17 (81)	36 (95)	0.09
INTERMACS Class 2, *n*(%)	6 (10)	4 (19)	2 (5)	0.2
SCAI stage D, *n* (%)	22 (37)	11 (52)	11 (29)	0.07
SCAI stage E, *n* (%)	37 (63)	10 (48)	27 (71)	0.07
Left ventricle ejection fraction,%	10 (0–20)	10 (0–23)	10 (0–19)	0.5
Right ventricle dysfunction, *n* (%)	24 (41)	8 (38)	16 (42)	0.7
Preserved right ventricular function, *n* (%)	35 (59)	13 (62)	14 (58)	0.7
Creatinine, mg/dl	2 (1.2–3.1)	1.7 (1.1–2.9)	2.3 (1.3–3.1)	0.2
Renal replacement therapy, *N* (%)	10 (17)	1 (5)	9 (40)	0.2
Total bilirubin, mg/dl	1 (0.9–2.4)	1.1 (0.9–1.6)	1.5 (1–2.6)	0.2
Platelets, number × mcL	146 (72–205)	160 (105–209)	133 (62–200)	0.4

Data are reported as median (interquartile).

MCS, mechanical circulatory support; VA-ECMO, venoarterial ECMO; INTERMACS, Interagency Registry for Mechanically Assisted Circulatory Support; SCAI, Society of Cardiovascular Angiography and Interventions.

**Table 3:** Revascularization procedure data.

Parameter	Total population (*n* = 42)	Recovery (*n* = 16)	No recovery (*n* = 26)	*p-*value
Revascularization, *n* (%)	37 (88)	15 (94)	22 (85)	0.4
Single-vessel disease, *n* (%)	14 (33)	10 (63)	4 (16)	0.003
Culprit lesion				
Left main/proximal LAD, *n* (%)	21 (50)	5 (31)	16 (62)	0.1
LAD, *n* (%)	13 (31)	8 (50)	5 (19)	0.047
Circumflex artery, *n* (%)	1 (2)	0 (0)	1 (4)	0.99
Right coronary, *n* (%)	3 (7)	2 (13)	1 (4)	0.5
LAD + Cx, *n* (%)	2 (5)	1 (6)	1 (4)	0.99
LAD + right coronary, *n* (%)	2 (5)	0 (0)	2 (7)	0.5
Complete vessel occlusion, *n* (%)	31 (74)	14 (88)	17 (65)	0.1
Complete revascularization, *n* (%)	22 (52)	12 (73)	10 (38)	0.02
Staged complete revascularization, *n*(%)	4 (10)	3 (19)	1 (4)	0.1
Troponine peak, ng/ml	9,603 (4,711–20,579)	9,289 (4,456–13,546)	9,603 (5,865–23,801)	0.4
Emergent coronary artery bypass grafting, *n* (%)	1 (2)	0 (0)	1 (4)	0.99
Successful revascularization procedure, *n* (%)	30 (71)	13 (80)	17 (65)	0.3
Patients undergoing coronary stenting, *n* (%)	30 (71)	13 (81)	17 (65)	0.3
Implanted stents, *n*	2 (1–3)	2 (1–3)	2 (1–4)	0.7
LAD/left main revascularization, *n*	38 (90)	14 (88)	24 (92)	0.6
Late presentation, *n*	19 (45)	4 (25)	15 (58)	0.057
TIMI 3, *n*	20 (48)	9 (56)	11 (42)	0.4

Data are reported as median (interquartile range) or number (percentage), as appropriate.

LAD, left anterior descending; Cx, circumflex; TIMI, thrombolysis in myocardial infarction.

All but one were implanted in the axillary artery (44 in the right axillary artery, 14 in the left axillary artery, and 1 in the right femoral artery for anatomical reasons). After Impella implantation, a cardiac index >2 lt/min/m^2^ was achieved in all patients in a few hours, and inotropic load was reduced in the first 48 h. Of note, the maximum inotropic score recorded during Impella 5.0/5.5 support was 15. Twenty-five percent of patients were weaned from inotropes within 72 h (Table 4) from Impella implantation, and all patients had no inotropic support at the time of MCS weaning. The left and right heart preload reduction to target values was reached within the first day of Impella 5.0/5.5 support in all patients. Native heart recovery was reached after a median support duration of 13 (8–20) days, with a maximum duration of 52 days. Device-related complications included major hemolysis in 11 patients (18%), of whom 7 underwent renal replacement therapy, 2 (3%) had mitral valve injury requiring interventional repair, and 2 (3%) underwent cerebrovascular accidents. Neither aortic valve damage was recorded, nor *de novo* aortic valve regurgitation (moderate or severe) was documented after Impella removal. Device exchange in the course of prolonged support was performed in nine patients due to device failure (15%): median Impella support in patients who experienced device exchange was 26 days. Axillary artery access was associated with insertion site complications: 4 patients (7%) needed surgery for site access bleeding, 2 patients (3%) experienced arm ischemia that was successfully treated, and 3 patients (5%) showed a transient deficiency of the brachiocephalic plexus.

**Table 4:** Characteristics of Impella support. Data are reported as median (interquartile range) or number (percentage), as appropriate.

	Total population (*n* = 59)	Recovery (*n* = 21)	No recovery (38)	*p*-value
Impella 5.0/5.5 support, days (IQR)	13 (8–20)	8 (12–15)	14 (8–24)	0.4
Mobilization with Impella, *n* (%)	23 (36)	11 (53)	12 (29)	0.1
Inotropes during Impella 5.0/5.5, days	7 (2–16)	5 (2–11)	8 (2–18)	0.9
Weaning from inotropes within 72 h from Impella 5.0/5.5 implant, *n*	15 (27%)	6 (29)	9 (26)	0.7
MV during Impella 5.0/5.5, days (IQR)	7 (3–14)	6 (1–9)	12 (5–17)	0.8
LV function improvement within 7–10 days after implant, *n* (%)	21 (36)	15 (71)	6 (16)	<0.001
NT-proBNP improving trend within 7–10 days after implant, *n* (%)	33 (56%)	11 (53%)	22 (58%)	0.7
Creatinine peak, mg/dl	3 (1.8–4.5)	2.3 (1.3–3.6)	3.2 (1.9–4.5)	0.1
Renal replacement therapy post Impella 5.0/5.5, *n* (%)	22 (36)	4 (19)	18 (47)	0.048
Bilirubin peak, mg/dl	2 (1.5–4.2)	1.8 (1.6–2.5)	3.2 (1.6–5.3)	0.05
CytoSorb, *n* (%)	28 (50)	9 (43)	19 (50)	0.6

VA-ECMO, venoarterial ECMO; MV, mechanical ventilation.

**Figure 2:**
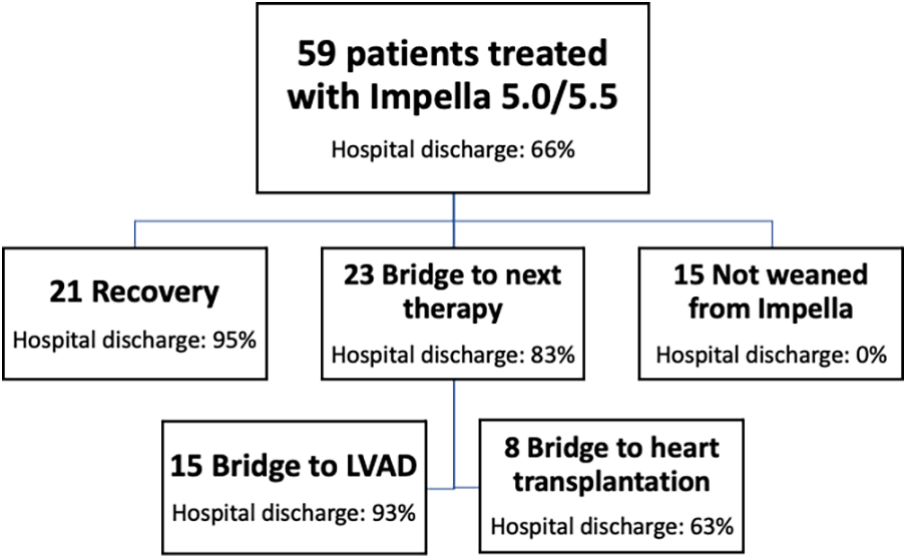
Study cohort evolution over three main pathways of care. tMCS, temporary mechanical circulatory support; GDMT, guideline-directed medical therapy.

The study cohort evolution through the different pathways of care (bridge to recovery, bridge to next therapy, or death before weaning) is shown in Figure 2. Twenty-one patients (36%) were weaned from MCS, while 21 patients could not be weaned: 15 patients (25%) were bridged to long-term MCS (LVAD), and 8 patients (14%) were listed for emergent heart transplantation (of whom 5 were successfully transplanted). Therefore, 44 out of 55 patients (75%) survived to next therapy or recovery, while 39 patients (66%) were discharged from the hospital (Figure 2). One LVAD patient died before hospital discharge due to non-reversible end-stage liver failure, while three of the patients listed for heart transplantation died on the waiting list: one for refractory cardiac arrest, one for multiorgan failure, and one had a fatal cerebral hemorrhage. One patient died after weaning from Impella due to sepsis. Fifteen patients (30%) died during Impella 5.0/5.5 support. For these patients, the cause of death was multiorgan failure in 9 patients (60%), refractory shock in 3 patients (20%), and other causes in the remaining 3 patients (20%). In patients weaned from MCS, LVEF at hospital discharge was 30 (24%–36%). The median follow-up time is 1,394 (443–1,660) days: patients with recovery had no readmissions for heart failure at the last available follow-up, which is more than 1 year in the vast majority of study patients.

Going deeper into data of the patients who experienced myocardial recovery, we observed a faster upgrade from another kind of tMCS to Impella 5.0/5.5 compared to patients who had no recovery of myocardial function [2(1–2) days vs. 3(1–7) days, *p* = 0.02]. The same was confirmed when looking specifically at Impella 5.0/5.5 implantation timing on top of VA-ECMO [2 (1–5) days in patients with recovery vs. 5 (2–11) days in the counterpart, *p* = 0.02] and when looking at Impella 5.0/5.5 implantation timing as an upgrade from another Impella device [2(2–2) vs. 5 (2–8), *p* = 0.06] (Table 2). No difference in recovery rate was observed between patients with resuscitation from cardiac arrest and those without. In patients with acute coronary syndrome (Table 3), single-vessel disease and complete revascularization were associated with myocardial recovery (63 vs. 16%, *p* = 0.003% and 73% vs. 38%, *p* = 0.02, respectively). The early presence (within 7–10 days from Impella implantation) of LV EF improvement, as documented by bedside echocardiography in the intensive care unit, was strongly associated with myocardial recovery (71% vs. 16%, *p* < 0.001) (Table 4). In patients with LV function improvement, LV EF increased from 10 (10–25)% to 25 (20–40)% after a few days of Impella support. In addition, patients successfully mobilized during Impella support were more in the recovery group compared to non-recovery patients (53% vs. 29%), although not statistically significant (Table 4). Overall, the degree of end-organ failure tended to be lower in patients with recovery compared to the others, as shown by a lower need for renal replacement (*p* = 0.048) and a trend toward lower bilirubin peak values (*p* = 0.05) (Table 4).

**Figure 3:**
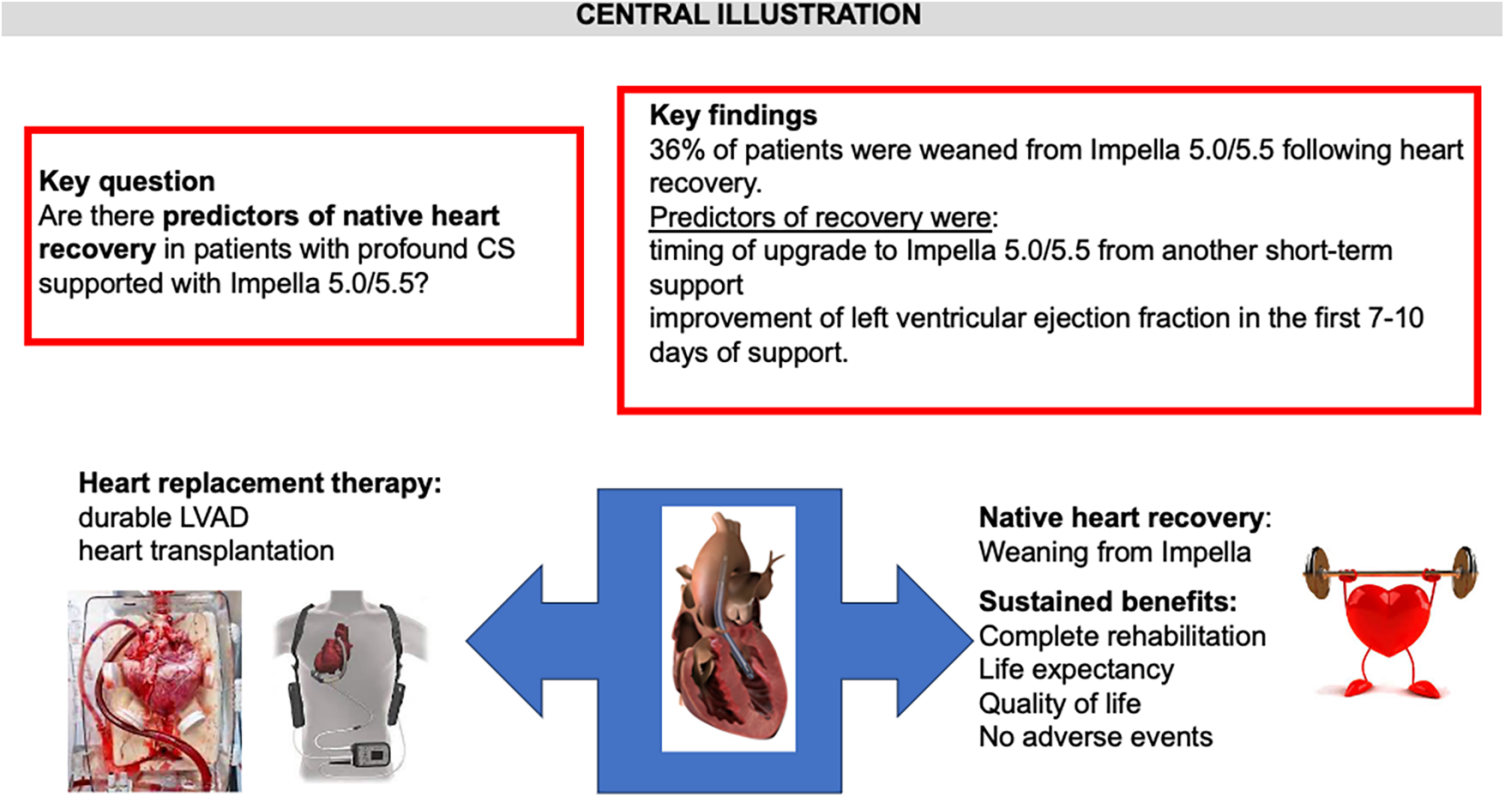
Central illustration that summarizes the key concepts of the manuscript.

Details of logistic and Fine and Gray regression univariate and multivariate analysis results are reported in the Supplementary Material. At Fine and Gray multivariate analysis, the number of days on tMCS before upgrade to Impella 5.0/5.5 [hazard ratio 0.68 (0.51–9) *p* = 0.0068] and improvement of LV EF within the first 7–10 days of support [hazard ratio 4.72 (1.34–16.7), *p* = 0.016] were found to be predictors of native heart recovery in patients supported with Impella 5.0/5.5 (see Supplementary Material and Figure 3). Preserved right ventricular function at presentation at Fine and Gray univariate regression analysis was not associated with the composite outcome of native heart recovery and/or survival (HR: 0.96 (0.38–2.44, *p* = 0.9365). Supplementary Figure S1 represents the trend of the cumulative incidence of the primary native heart recovery event and competitive risks, throughout the follow-up.

## Discussion

New-onset heart failure is often a rapidly evolving clinical condition at risk of hemodynamic deterioration. Minimal invasive LVADs are emerging as game changers for patients with new-onset acute heart failure where the evaluation of the clinical case, efficacy of the urgent procedure, evolution of the primary disease and organ function, and perspective of recovery are all to be addressed during the treatment of CS. Impella 5.0/5.5 gives the patient the opportunity for immediate survival and the clinicians the opportunity to decide the appropriate final treatment. In particular, transaortic pVADs that can be implanted through axillary access greatly innovated the approach to patients on short-term MCS, since they enable early patients' extubation and complete rehabilitation (25–27).

This study currently represents the largest experience from South Europe on the use of this technology for the treatment of deep cardiogenic shock of different origins. While recent studies on Impella have addressed predictors of survival (27), of note, this is the first study that could identify predictors of native heart recovery in patients supported with Impella 5.0/5.5 devices.

Our data are in line with existing literature (11–18, 25–27) and confirm the efficacy and safety of minimal invasive LVADs in such critical care scenarios, even for prolonged support duration (up to 52 days). They are able to fully support the patients up to weeks through different pathways of care. Namely, patients on temporary LVAD, navigate up to three possible destinies: (1) weaning from MCS for myocardial recovery; (2) bridge to heart substitution (i.e., long-term LVAD vs. heart transplantation); and (3) irreversible derangements of shock and organ damage. All these pathways are demanding for the patients and hospitals' systems, both in terms of clinical and economic resources. We put specific emphasis on recovery as it represents the most desirable clinical outcome. With respect to this, evidence that full mechanical unloading of the heart is crucial to promote recovery is accumulating: this process, however, requires time since it involves several cellular mechanisms (20), making immediate recovery after the acute phase very rare. Technical characteristics of pVADs open the way to a weaning process that appears more attractive than for other types of MCS, since pVADs embody the concept of full mechanical unloading to promote myocardial recovery and viability with intact cardiac structures (no apical coring). In our population, myocardial recovery with weaning from MCS and no inotropic therapy was observed in 36% of patients, in line with what was recently reported in the European (17) and US (16–18) series of similar patients treated with the same devices. Currently, it is extremely complex at baseline to foresee which patients will experience myocardial recovery, beyond the setting of acute myocarditis, which presents higher recovery chances. This issue is nevertheless of paramount importance for clinical practice: ensuring the most proficuous pattern for each patient is pivotal but not straightforward due to the complexity of the organ allocation system on one side and the burden for the society represented by LVAD therapy on the other side. Hemodynamic monitoring tools and echocardiography currently represent the cornerstone of monitoring, but few parameters of these tools have been validated in critically ill patients supported by tMCS. As an example, cutoff values and reasoning from classical (stable) cardiology and heart failure are applied to CS patients, but data regarding these values are often lacking, making the management of these patients more complex for clinicians (28). We tried to identify a few simple parameters that can be evaluated at clinical presentation and in the early phase of MCS with Impella 5.0/5.5 and may help to prognosticate recovery. Our data showed that time on tMCS before upgrade to Impella 5.0/5.5 and the early presence of LVEF echocardiographic improvement (within the first 7–10 days of MCS) were predictors of recovery at multivariate analysis. These findings might be relevant for clinical practice, because they may orient clinical decision-making towards action that may improve patients' recovery chances (for example, the earlier upgrade of tMCS). On the other hand, LVEF proved to be a predictor of recovery in this population: patients who did not present signs of LVEF improvement within the first 7–10 days of support presented a lower rate of successful Impella weaning in our experience. For these patients, a bridge to further therapeutic strategy, either toward long-term MCS or heart transplantation, might be tested in this time frame. Indeed, bridging LVAD in the most favorable clinical conditions presents better outcomes and may reduce adverse events and the complexity of ICU and hospital stay (9). In light of this, patients on transaortic LVAD were hemodynamically evaluated for RV function, pulmonary hypertension, and candidacy to LVAD/heart transplantation and were treated to reach spontaneous breathing, oral feeding, and active mobilization. Although burdened by the issues of echocardiography assessment in CS patients on tMCS (28), where the applicability of standard echocardiographic parameters both for left and right ventricle is not straightforward, the evidence of LV EF improvement within the first days of treatment is in support of past preliminary evidence that improvement of the cardiac index is associated with higher chances of heart recovery in postcardiotomy CS patients supported with pVAD (29). This consideration stresses the importance not only of baseline patients’ characteristics but also of the early response to treatment, as a key element to be taken into account with respect to native heart recovery chances.

The crucial importance of myocardial recovery promotion and prediction in acute cardiogenic shock is clear also to the scientific community, and it is currently a major focus of translational scientific research (20). Technology development will arguably evolve into algorithms of the MCS devices that may help in the complex process of weaning, making it automatic and autonomic. However, it should be underlined that the concept of MCS weaning is extremely complex and inseparably linked to the “weaning of the patient from critical status,” not only to the weaning from the device. In achieving this goal in clinical practice, multiple factors (primary disease, interventional procedures, adverse events) may concur to prolong the time of MCS that will permit recovery of the patient who will then experience recovery of the heart and return to normal life with no major sequelae. Since after durable LVAD, myocardial recovery can be expected in <5% of patients according to large statistics of unselected LVAD patients (30, 31), the new optimal window to test native heart recovery is the period on Impella support in the journey of CS complicating heart failure.

Assessment of native heart recovery also implies right ventricular function evaluation, which is not straightforward in the case of prolonged LV support, as for patients included in the present study. Although we could not find any significant statistical association, it is imperative to stress the importance of this topic, which should be a target also for future larger studies on more homogeneous populations.

This study provides preliminary data on a very complex population and, as such, presents some limitations. First of all, the observational design and the fact that patients were treated at a single center may limit the validity of the findings. Furthermore, we were able to identify relevant clinical parameters associated with myocardial recovery and MCS weaning, but we are fully aware that the identified predictors of native heart recovery should be further tested in larger populations. The heterogeneity of our study population, where different etiologies of CS were present (viz., with both AMI and non-AMI CS patients), may have influenced our analysis and might have affected the results. Furthermore, different etiologies of CS may also present different recovery targets. However, the study population reflected the real clinical world scenario of CS, and, as such, data presented in our study are useful for clinicians dealing with CS patients. In addition, the vast majority of our patients were “*de novo*” CS patients at the first episode of heart failure. Similarly, we are aware that patients who received Impella 5.0/5.5 support in the context of therapy escalation were different from patients who underwent therapy de-escalation through Impella. Again, this element can be ascribed to the consideration that the present study reported original data from contemporary clinical practice scenarios with an all-comers approach: since the focus of this study is the prolonged Impella support and patients' pathways of care, this element is not expected to initiate the findings of the study. We also acknowledge that the present study is underpowered to provide robust evidence on predictors of native heart recovery. Furthermore, we included among predictor even parameters measured after Impella implantation: although supported by literature (29), we agree that this approach complicates patients’ stratification at the time of Impella implantation with respect to recovery chances. As a result, the value and the current implications of our findings, due to the abovementioned study limitations, remain limited for contemporary clinical practice. Now that preliminary data suggest, however, that the recovery prognostication might be possible in patients on tMCS with Impella, larger studies to analyze predictors of myocardial recovery on Impella support with robust statistical methodology are warranted.

In conclusion, Impella 5.0 and 5.5 are key devices for the treatment of profound cardiogenic shock, not limited to survival outcomes. Patients navigate through different pathways of care: low invasiveness is a key factor that simplifies patients' recovery. We provided preliminary evidence that the identification of myocardial recovery predictors at presentation and in the early phase of support might be possible and is expected to be useful to improve patients' management, namely, promoting heart recovery where possible vs. speeding up heart transplantation listing or durable LVAD implantation. Larger prospective studies are ultimately warranted to identify predictors of native heart recovery in homogenous populations of CS of similar etiology.

## Data Availability

The original contributions presented in the study are included in the article/**Supplementary Material**; further inquiries can be directed to the corresponding author.
