# Supplementary material for: Prolonged Impella 5.0/5.5 support within different pathways of care for cardiogenic shock: the experience of a referral center
Source: Front Cardiovasc Med. 2024 Jul 2;11:1379199. doi: 10.3389/fcvm.2024.1379199 (PMC11250607; doi:10.3389/fcvm.2024.1379199)

**Supplementary material**

**Table 1. Univariate logistic regression analysis for parameters associated with native heart recovery.** Only significant outcomes of the variables included in the univariate analysis.

| **Variable** | **OR (95% CI)** | **P-value** |
| --- | --- | --- |
| Chronic heart failure | 3.09 (0.87-10.96) | 0.0805 |
| Complete Revascularization | 0.044 (0.003-0.662) | 0.0239 |
| Late Revascularization | 4.4 (1.09-17.72) | 0.0371 |
| Days of MCS pre-impella | 0.82 (0.68-0.99) | 0.0476 |
| Days of ECMO pre-impella | 0.7 (0.47-1.07) | 0.1042 |
| Days of impella percutaneous pre-Impella 5.0/5.5 | 0.83 (0.66-1.04) | 0.1020 |
| INTERMACS Class 2 | 0.22 (0.04-1.34) | 0.101 |
| SCAI class E | 2.7 (0.89-8.17) | 0.0786 |
| Preserved right ventricular function | 1.18 (0.4-3.52) | 0.7641 |
| Continuous renal replacement therapy | 6.11 (0.71-52.25) | 0.0985 |
| Mobilization with impella | 0.38 (0.124-1.152) | 0.0871 |
| Max bilirubine | 0.79 (0.61-1.03) | 0.0852 |
| Hospital Survival | 0.05 (0.006-0.41) | 0.0053 |
| Survival 28 days | 0.03 (0.002-0.59) | 0.0208 |
| Improvement LV EF within 7-10 days from impella start | 0.053 (0.013-0.22) | <.0001 |
| Single vesses disease | 0.1 (0.02-0.46) | 0.0030 |

OR – Odds Ratio - MCS – mechanical circulatory support, ECMO – extracorporeal membrane oxygenation, INTERMACS - interagency registry for mechanically assisted circulatory support, SCAI - Society of cardiovascular angiography and interventions LV EF – left ventricular ejection fraction.

**Table 2. Results of multivariate logistic regression analysis to identify predictors of native heart recovery.**

| **Variabile** | **OR (95% CI)** | **P-value** |
| --- | --- | --- |
| Log [Days of MCS pre-impella] | 0.1 (0.02-0.46) | 0.0032 |
| Improvement LV EF within 7-10 days from impella start | 56.8 (6.81-423.33) | 0.0002 |

OR – Odds Ratio, MCS – mechanical circulatory support

**Table 3. Univariate Fine and Grey regression analysis for parameters associated with native heart recovery in competing risk outcomes.** Only significant outcomes of the variables included in the univariate analysis.

| **Variable** | **HR (95% CI)** | **P-value** |
| --- | --- | --- |
| Chronic heart failure | 0.52 (0.17-1.58) | 0.2487 |
| Complete Revascularization | 5.6 (1.35-23.2) | 0.0176 |
| Late Revascularization | 0.38 (0.14-1.01) | 0.0530 |
| Days of MCS pre-impella | 0.80 (0.63-0.98) | 0.0306 |
| Days of ECMO pre-impella | 0.69 (0.51-0.95) | 0.0216 |
| Days of impella percutaneous pre-Impella 5.0/5.5 | 0.81 (0.62-1.05) | 0.1081 |
| INTERMACS Class 2 | 1.77 (0.6-5.23) | 0.3026 |
| SCAI class E | 0.46 (0.21- 1.04) | 0.0625 |
| Preserved right ventricular function | 0.96 (0.38-2.44) | 0.9365 |
| Continuous renal replacement therapy | 0.15 (0.03-0.8) | 0.0261 |
| Mobilization with impella | 0.91 (0.39-2.14) | 0.8254 |
| Max bilirubine | 0.85 (0.703-1.03) | 0.0905 |
| Hospital Survival | 14.6 (2.75-77.96) | 0.0017 |
| Improvement LV EF within 7-10 days from impella start | 4.56 (1.6-13) | 0.0055 |
| Single vesses disease (Ref=0) | 4.02 (1.45-11.15) | 0.0075 |

HR – Hazard Ratio - MCS – mechanical circulatory support, ECMO – extracorporeal membrane oxygenation, INTERMACS - interagency registry for mechanically assisted circulatory support, SCAI - Society of cardiovascular angiography and interventions LV EF – left ventricular ejection fraction.

**Table 4. Results of multivariate Fine and Grey analysis to identify predictors of native heart recovery in competing risk outcomes.**

| **Variabile** | **HR (95% CI)** | **P-value** |
| --- | --- | --- |
| Days of MCS pre-impella | 0.68 (0.51-0.9) | 0.0068 |
| Improvement LV EF within 7-10 days from impella start | 4.72 (1.34-16.7) | 0.016 |

HR – Hazard Ratio, MCS – mechanical circulatory support, LV EF – left ventricular ejection fraction

**Figure 1.** Cumulative incidence of native heart recovery, with mortality, heart transplant (HTx) and durable left ventricular assist device (LVAD) as competing risk


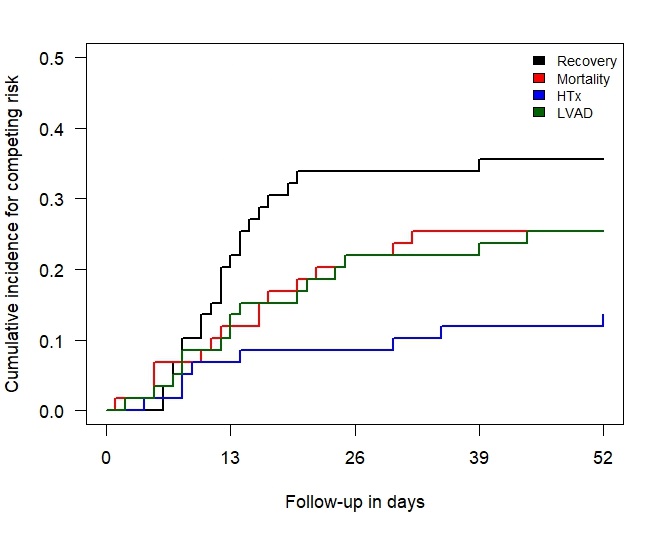

Supplement: Supplementary file 1 [file Table1.docx]
